# Supplementary material for: Improving the fast-charging capability of NbWO-based Li-ion batteries
Source: Nat Commun. 2025 Mar 11;16:2441. doi: 10.1038/s41467-025-57576-1 (PMC11897329; doi:10.1038/s41467-025-57576-1)
Supplement: Supplementary file 1 — Supplementary Information [file 41467_2025_57576_MOESM1_ESM.pdf]

Supplementary Information

Improving the fast-charging capability of NbWO-based Li-ion batteries

Yaqing Guo[1]†, Chi Guo[2]†, Penghui Li[3]†, Wenjun Song[1], Weiyuan Huang[4], Junxin Yan[3], Xiaobin Liao[5], Kun He[1,*], Wuxin Sha[6], Xuemei Zeng[1], Xinyue Tang[1], QingQing Ren[1], Shun Wang[1], Khalil Amine[4], Anmin Nie[3,*], Tongchao Liu[4,*], Yifei Yuan[1,*]

[1]College of Chemistry and Materials Engineering, Wenzhou University, Wenzhou, 325035, China

[2]Jiangsu Key Laboratory for Design and Manufacture of Micro-Nano Biomedical Instruments, School of Mechanical Engineering, Southeast University, Nanjing, 211189, China

[3]Center for High-Pressure Science, State Key Laboratory of Metastable Materials Science and Technology, Yanshan University, Qinhuangdao, 066004, China

[4]Chemical Sciences and Engineering Division, Argonne National Laboratory, Lemont, IL, 60439 USA

[5]State Key Laboratory of Advanced Technology for Materials Synthesis and Processing International School of Materials Science and Engineering, Wuhan University of Technology Wuhan, 430070, China

[6]State Key Laboratory of Advanced Electromagnetic Engineering and Technology, School of Electrical and Electronic Engineering, Huazhong University of Science and Technology, Wuhan, 430074

†These authors contributed equally to this work.

*Correspondence: yifeiyuan@wzu.edu.cn (Prof. Yuan); liut@anl.gov (Prof. Liu); anmin@ysu.edu.cn (Prof. Nie); hekun@wzu.edu.cn (Prof. He)

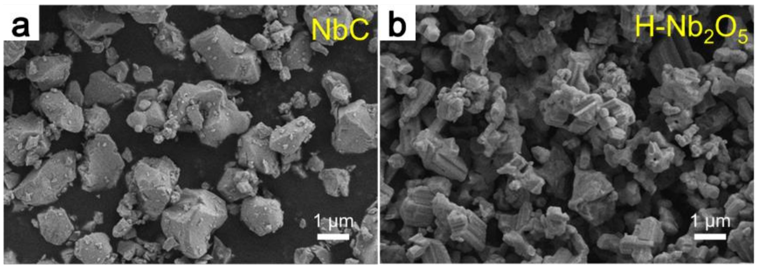

Supplementary Fig.1. The SEM images of a) NbC, b) H-Nb_2O_5.

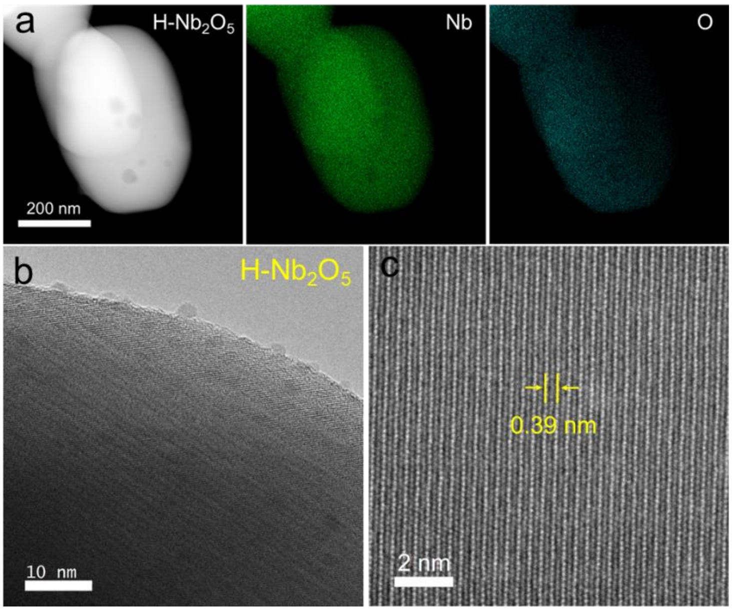

Supplementary Fig. 2. a) The corresponding EDS element mappings of H-Nb_2O_5. b) and c) High-resolution HAADF STEM images of H-Nb_2O_5.

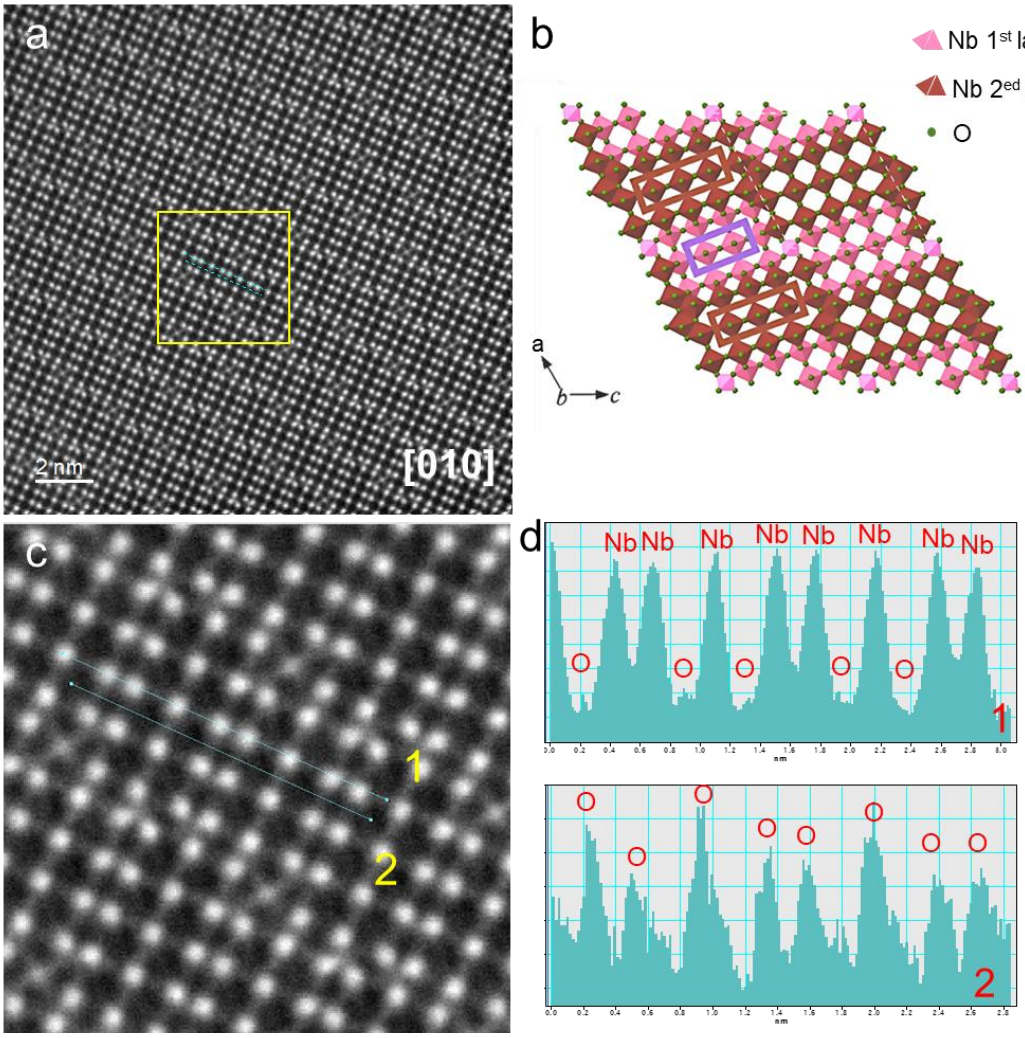

Supplementary Fig. 3. a) HAADF STEM images of H-Nb_2O_5 at [010] zone axis. b) Schematic of H-Nb_2O_5 crystallographic shear structure along b axis. c) Enlarged view of the yellow region in a) and d) the corresponding line profile.

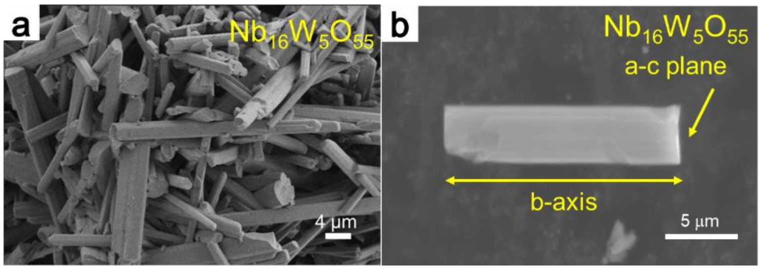

Supplementary Fig. 4. The SEM images of a) $Nb_{16}W_5O_{55}$ and b) single $Nb_{16}W_5O_{55}$ crystal.

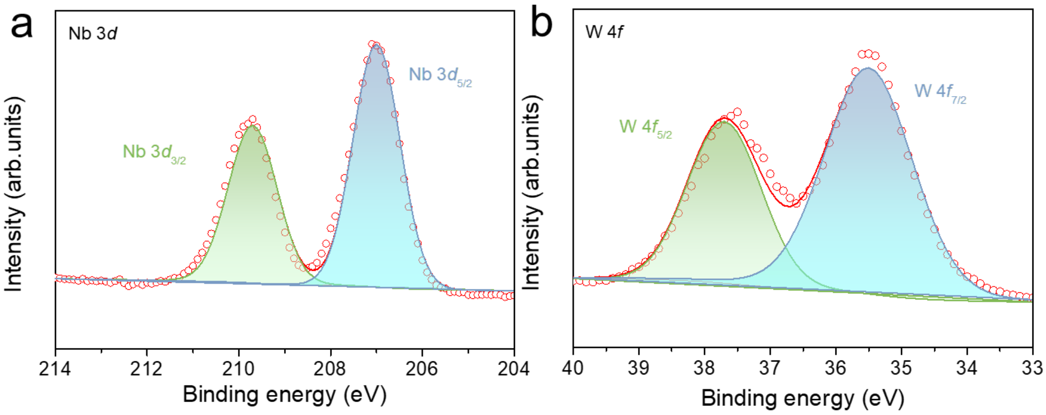

Supplementary Fig. 5. The XPS spectra of a) Nb 3*d*, and b) W 4*f* for $Nb_{16}W_5O_{55}$. The intensity is derived from the measured counts and normalized to the maximum value for comparison.

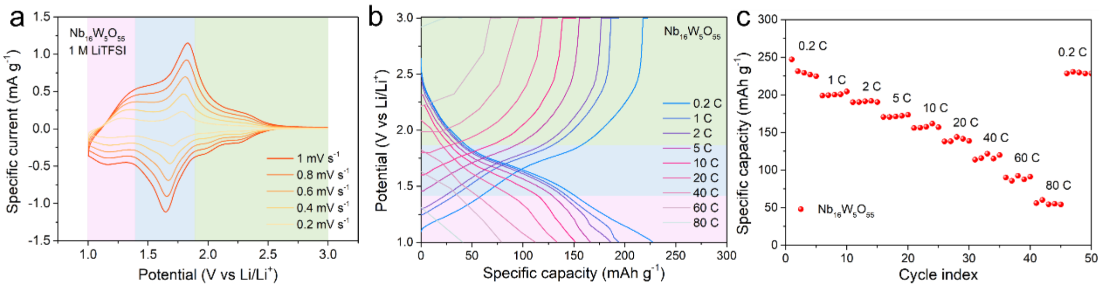

Supplementary Fig. 6. The a) CV curves, b) charging/discharging curves, and c) rate performance of $Nb_{16}W_5O_{55}$ in 1 M LiTFSI.

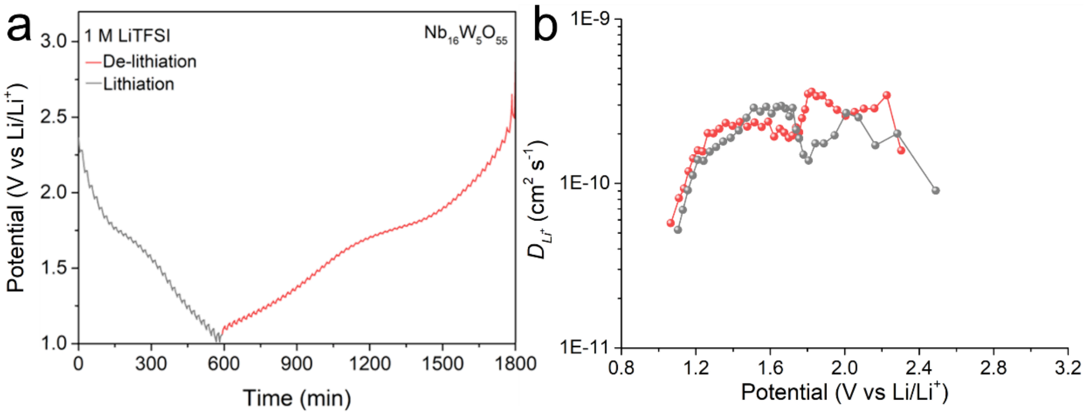

Supplementary Fig. 7. The GITT curves and the chemical diffusion coefficients of Li^+ (D_{Li^+}) plots for $Nb_{16}W_5O_{55}$.

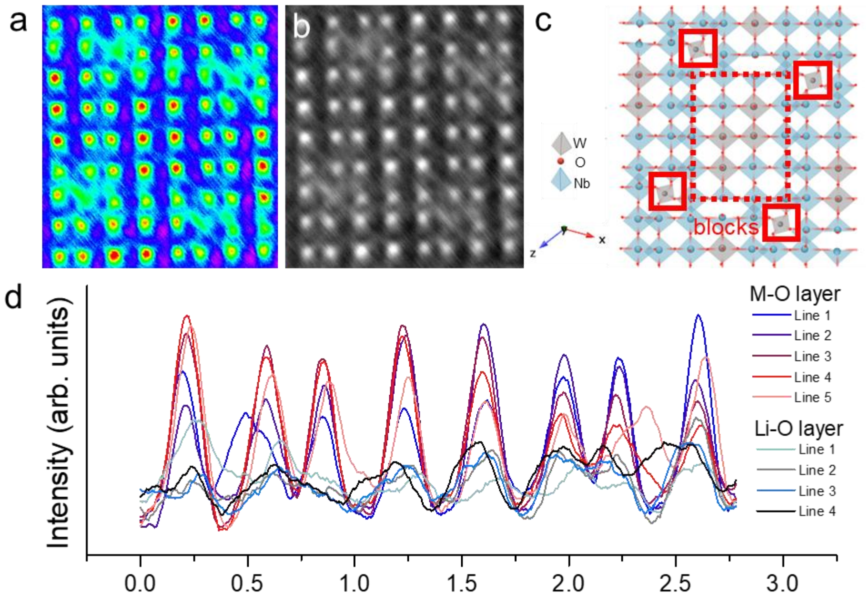

Supplementary Fig. 8. ABF macrographs of a) the pristine $Nb_{16}W_5O_{55}$ (the images are presented in reverse color) and b) the inverted ABF image contrast at [010] zone axis. c) Crystal structure of pristine $Nb_{16}W_5O_{55}$ viewing along [010] zone axis. d) Intensity line profile along the arrows indicated in Figure 2a. The Y-axis value for the basis line reflects the intensity of the darkest location within the area of interest, X-axis with the unit of nm.

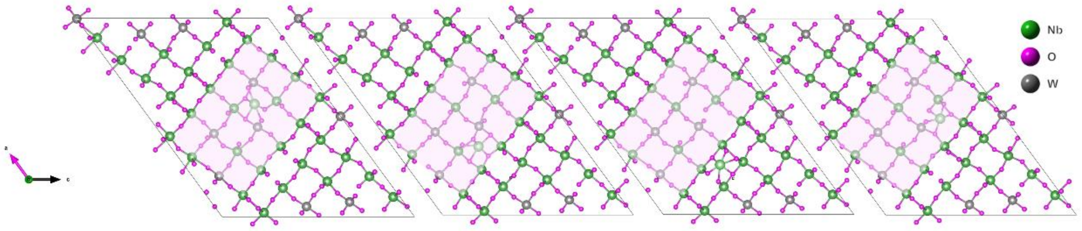

Supplementary Fig. 9. Calculated schematic pictures of the $Nb_{16}W_5O_{55}$-lattice after Li-ion intercalation.

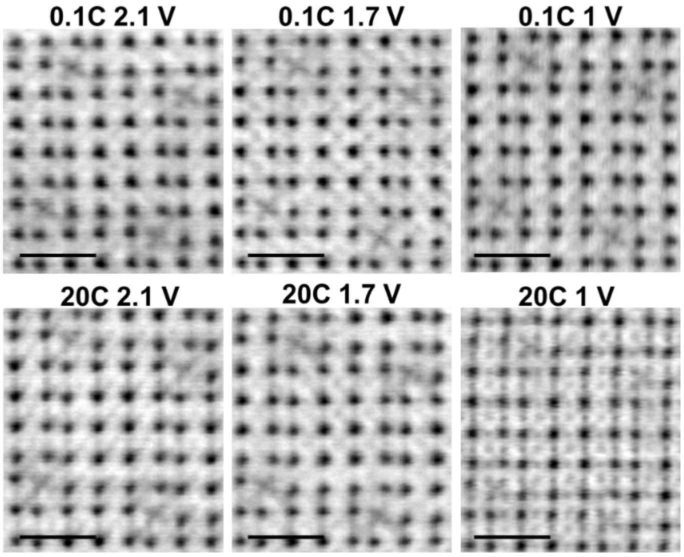

Supplementary Fig. 10. ABF STEM images of $Nb_{16}W_5O_{55}$ at [010] zone axis at 0.1 C and 20 C at 2.1 V, 1.7 V, and 1 V, respectively (scale bar is 1 nm).

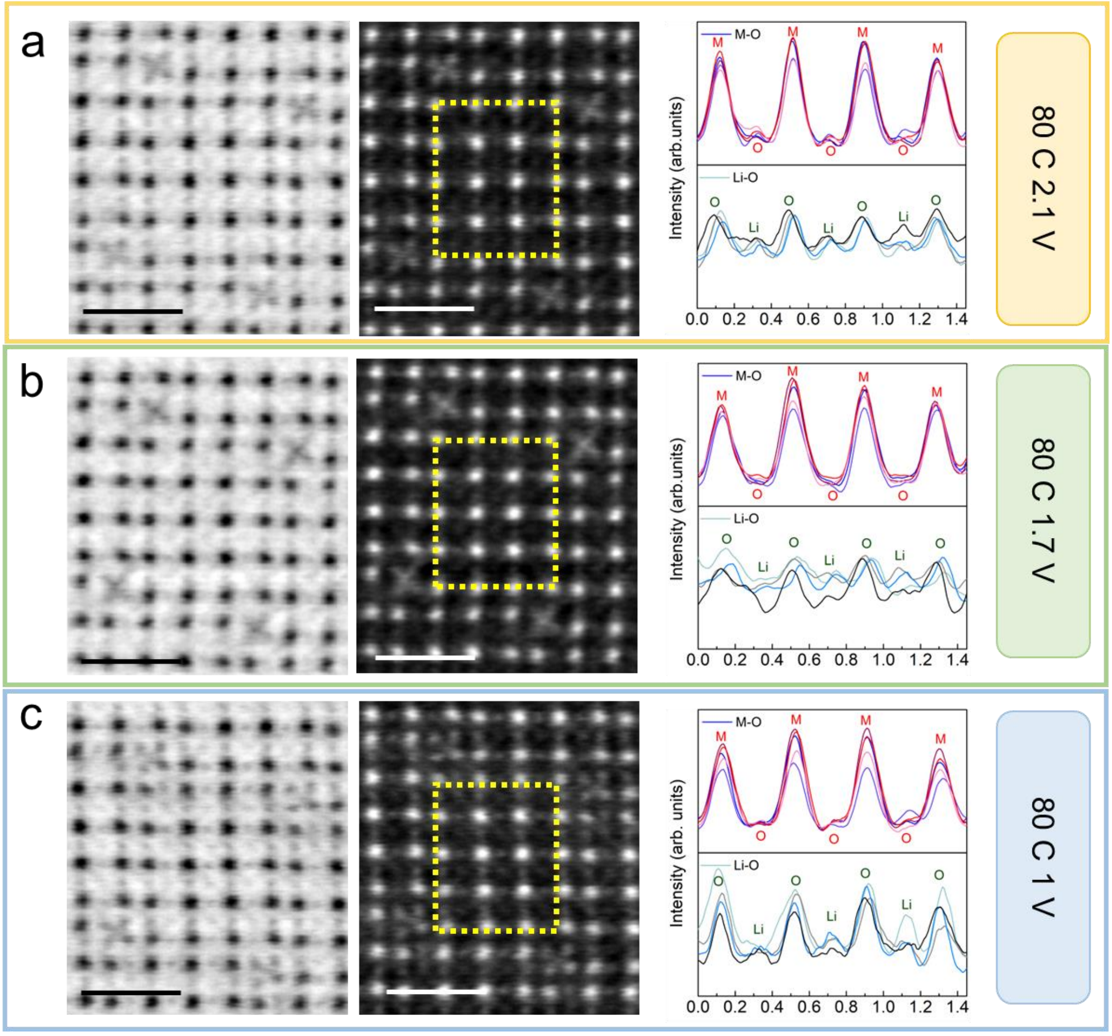

Supplementary Fig. 11. The ABF images, corresponding reverse color images and line profile for $Nb_{16}W_5O_{55}$ discharge with the rate of 80 C at a) 2.1 V, b) 1.7 V, and c) 1 V, respectively (scale bar is 1 nm). The Y-axis value for the basis line reflects the intensity of the darkest location within the area of interest, X-axis with the unit of nm.

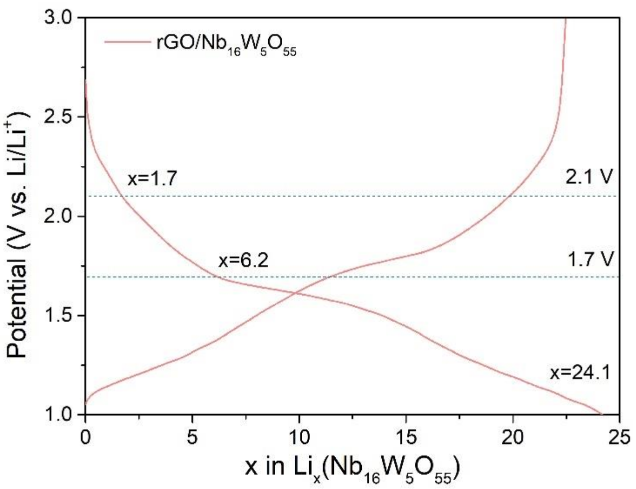

Supplementary Fig. 12. Lithium-ion content at different voltages with a rate of 0.1 C.

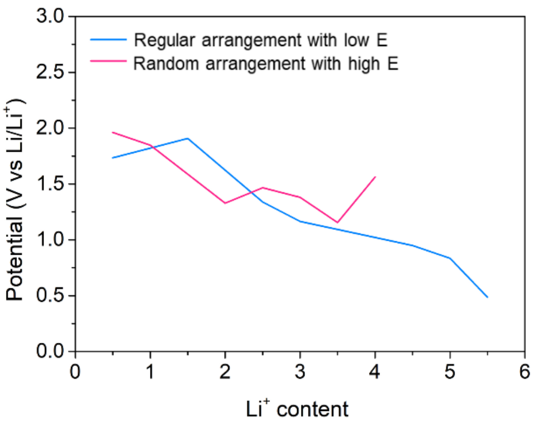

Supplementary Fig. 13. The DFT predictions for the voltage profile at different intercalation models. Low E represents the state with the lowest energy for lithium-ion intercalation[1], while high E refers to a high-energy state caused by random intercalation.

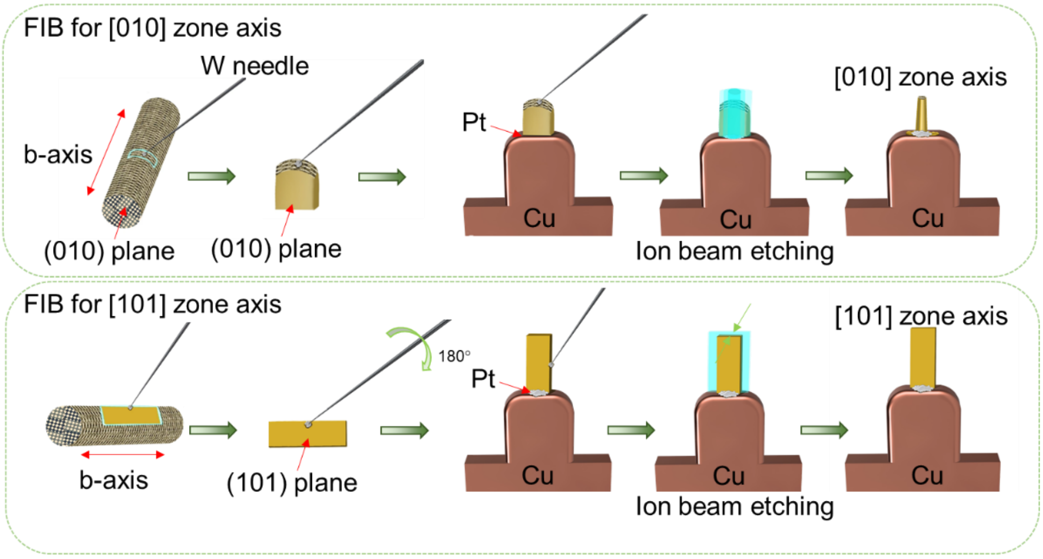

Supplementary Fig. 14. Schematic of FIB for preparing samples of rGO/$Nb_{16}W_5O_{55}$ with [010] and [101] zone axes.

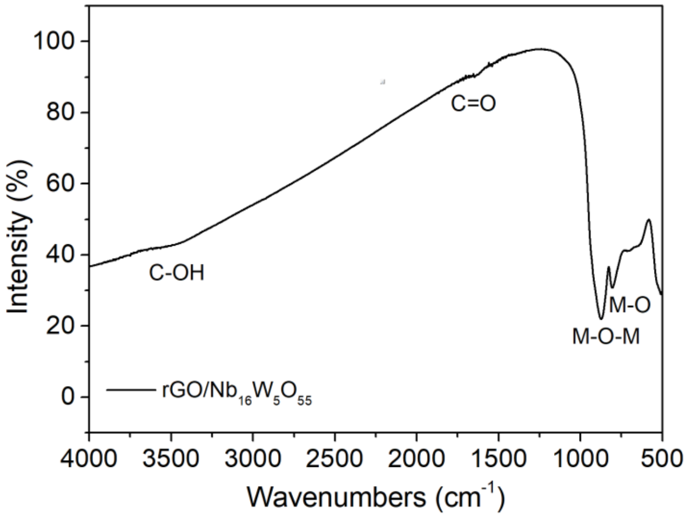

Supplementary Fig. 15. The FTIR spectrum of rGO/$Nb_{16}W_5O_{55}$ (M = Nb or W). The intensity is calculated from the measured counts and then normalized to a scale of 0 to 100%, with the maximum value set as 100% for comparison.

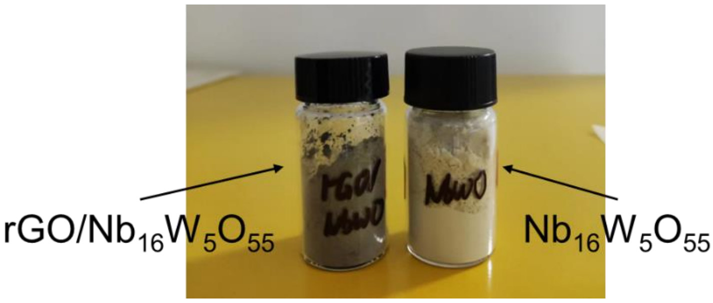

Supplementary Fig. 16. Pictures of $Nb_{16}W_5O_{55}$ and rGO/$Nb_{16}W_5O_{55}$ powders.

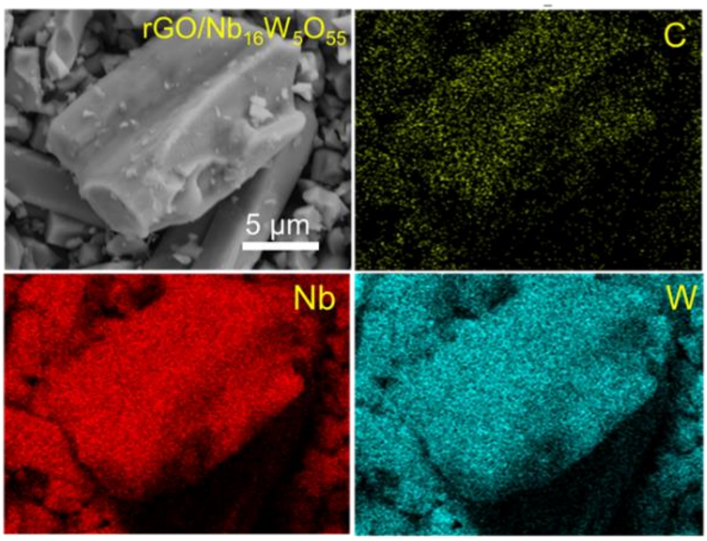

Supplementary Fig. 17. SEM image of rGO/$Nb_{16}W_5O_{55}$ and the corresponding EDS element mappings of C, Nb and W.

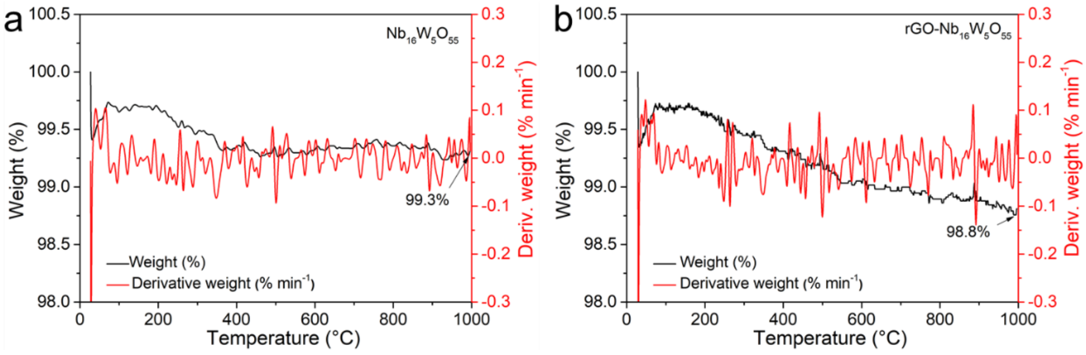

Supplementary Fig. 18. TG-DSC curves of a) $Nb_{16}W_5O_{55}$, and b) rGO/$Nb_{16}W_5O_{55}$ in the air.

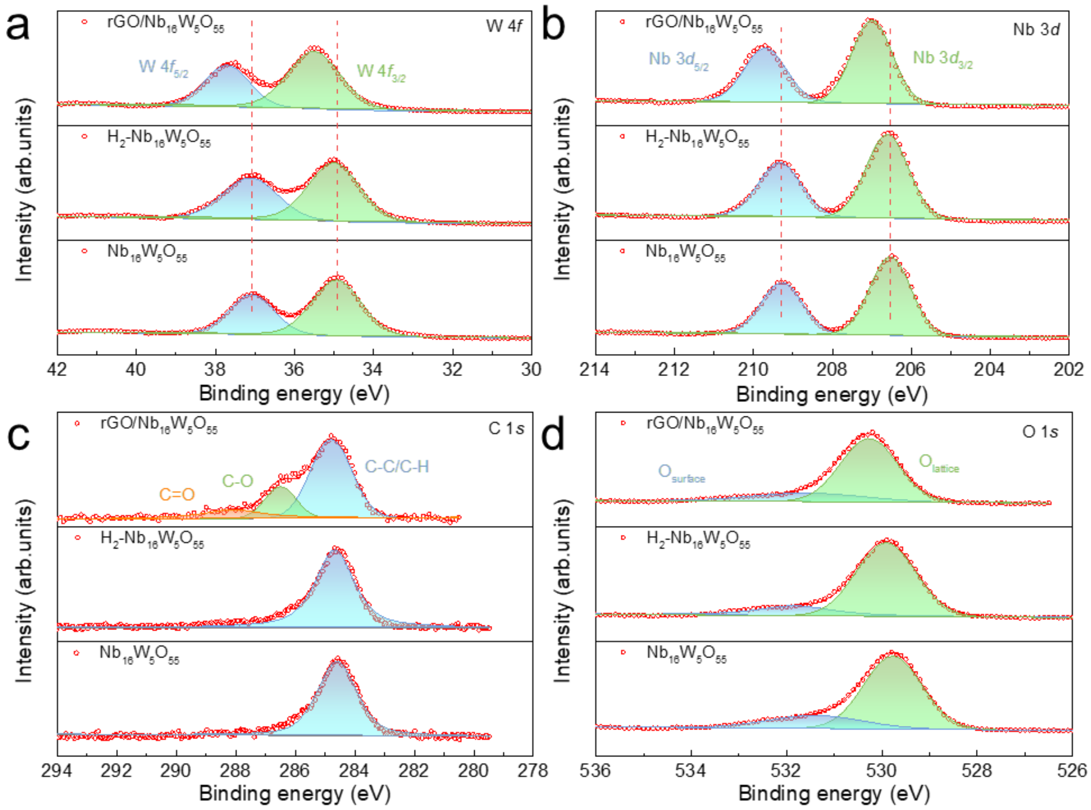

Supplementary Fig. 19. The XPS spectra of a) W 4*f*, b) Nb 3*d*, c) C 1*s* and d) O 1*s* for $Nb_{16}W_5O_{55}$, H_2-$Nb_{16}W_5O_{55}$ and rGO/$Nb_{16}W_5O_{55}$, respectively. The intensity is derived from the measured counts and normalized to the maximum value for comparison.

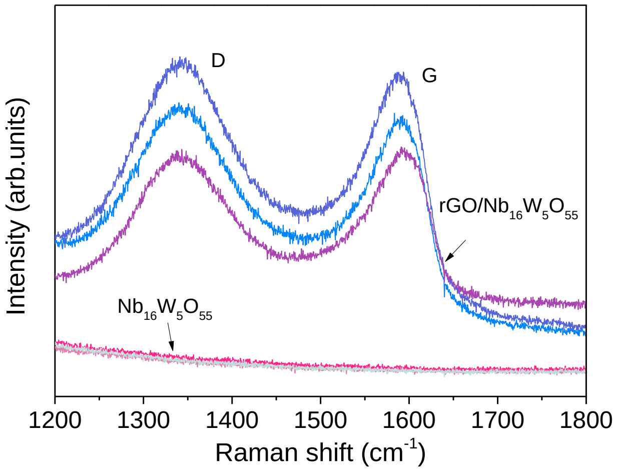

Supplementary Fig. 20. The Raman spectra of $Nb_{16}W_5O_{55}$ and rGO/$Nb_{16}W_5O_{55}$ (each material was tested three times). The intensity is derived from the measured counts and normalized to the maximum value for comparison.

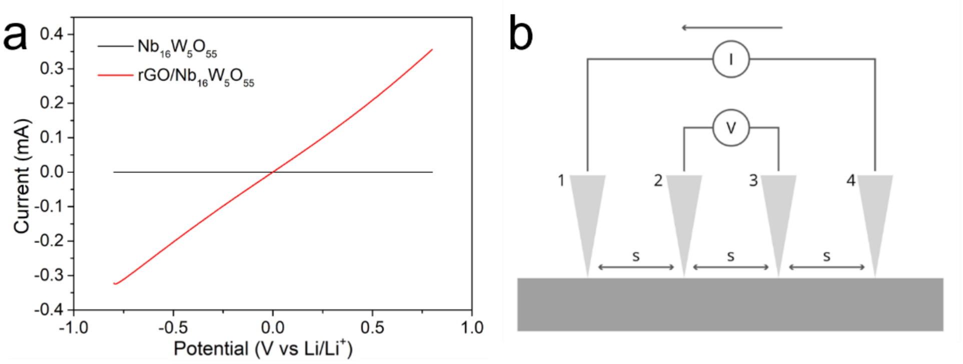

Supplementary Fig. 21. a) The *I*-*V* curves of $Nb_{16}W_5O_{55}$ and rGO/$Nb_{16}W_5O_{55}$ pressed pellets tested using the four-probe method based on cryogenics probe station and tested by autolab 302N. b) The resistivity equation can be simplified as $\rho=\Delta V_{23} \cdot 2\pi s/I$, where s is the spacing between two probes, I is the applied current, and ΔV_{23} is the voltage difference between probes 2 and 3. The values ΔV_{23} and I are the measurement result from the autolab 302N, which is output directly by the testing instrument.

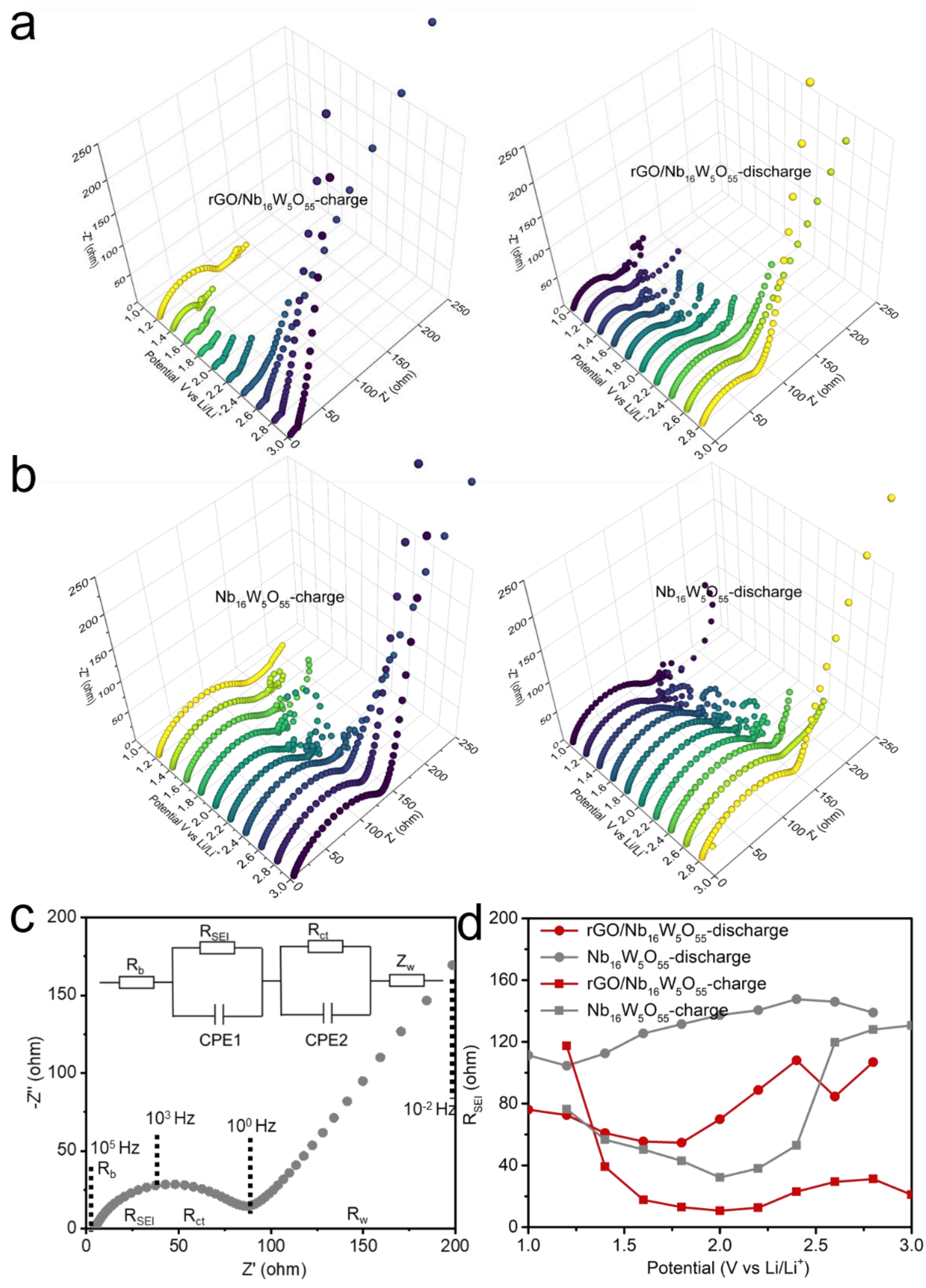

Supplementary Fig. 22. *In situ* EIS for a) rGO/$Nb_{16}W_5O_{55}$ and b) $Nb_{16}W_5O_{55}$ over a voltage range of 1 V to 3 V (frequency range: 0.01 Hz~100000 Hz). c) Equivalent circuit for fitting. d) Fitted values of impedance at different voltages.

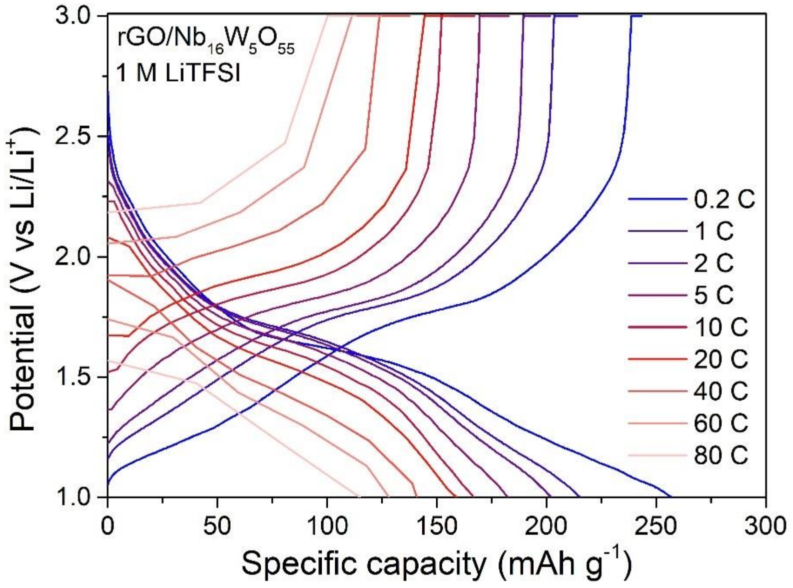

Supplementary Fig. 23. The charging/discharging curves of rGO/$Nb_{16}W_5O_{55}$ in 1 M LiTFSI.

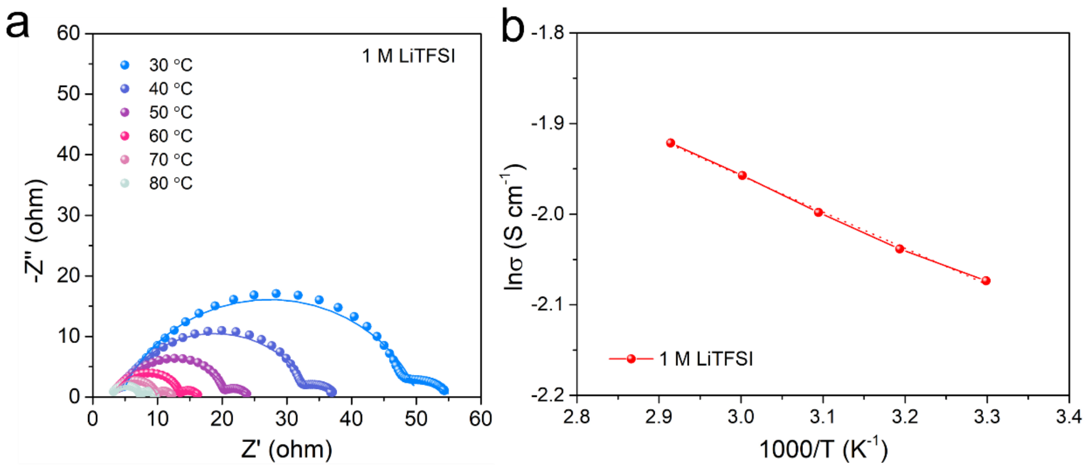

Supplementary Fig. 24. a) The EIS curves for Li||Li symmetrical cell and b) the temperature dependence curves of the ionic conductivities for different electrolytes based on stainless steel||stainless steel symmetrical cell for 1 M LiTFSI.

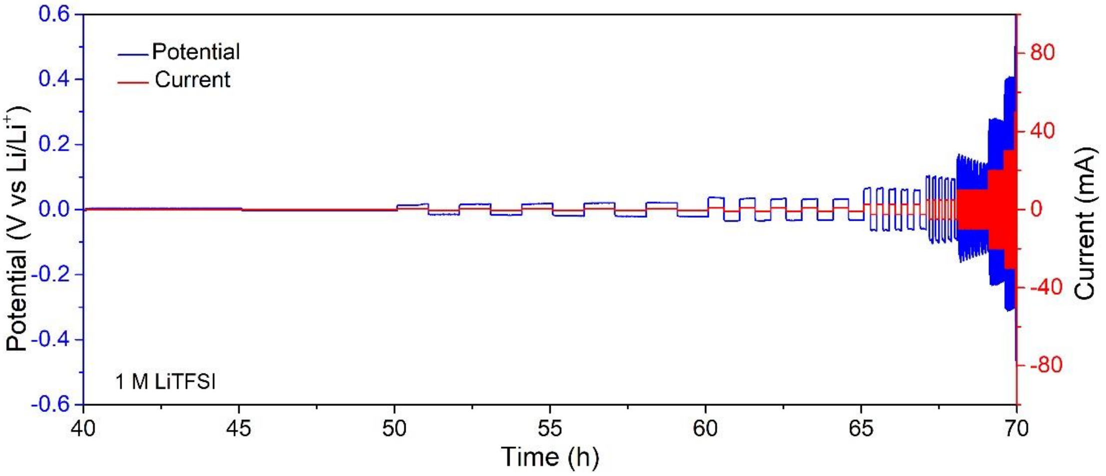

Supplementary Fig. 25. Rate performance of Li||Li symmetric cell.

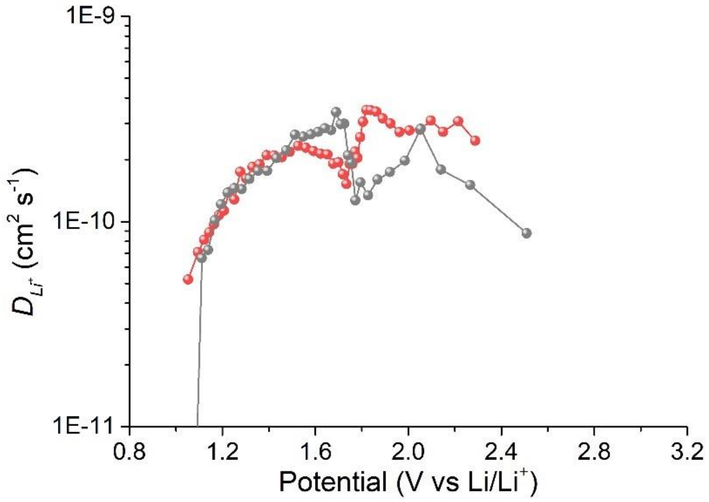

Supplementary Fig. 26. The chemical diffusion coefficients of Li^+ (D_{Li^+}) plots for rGO/$Nb_{16}W_5O_{55}$ from Fig. 5f.

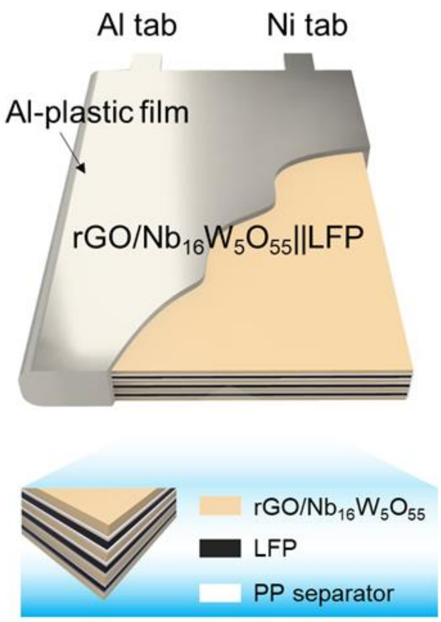

Supplementary Fig. 27. Schematic diagram of pouch cell.

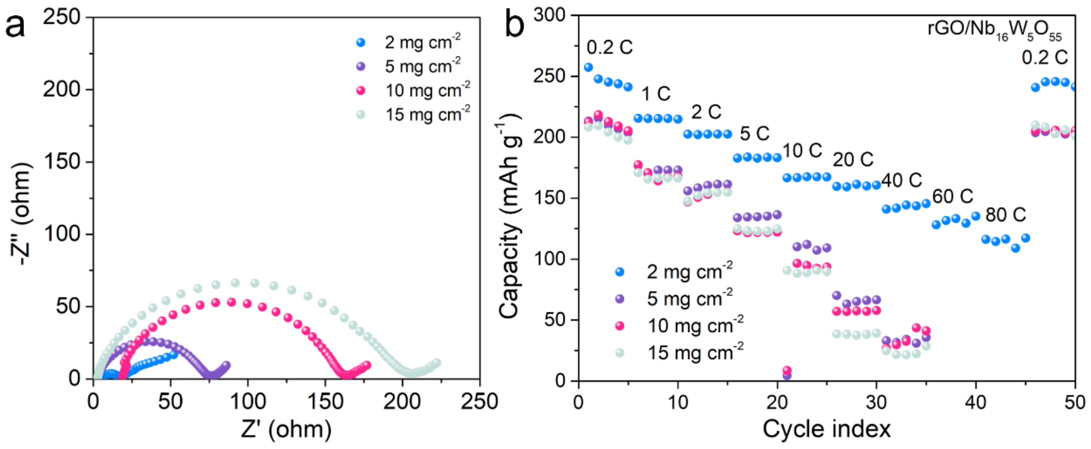

Supplementary Fig. 28. The a) EIS and b) the rate performance of rGO/$Nb_{16}W_5O_{55}$ with different mass loading. Due to the limitations of the instrument's range, lithium-ion batteries with high load capacities cannot be tested for ultrahigh-rate performance.

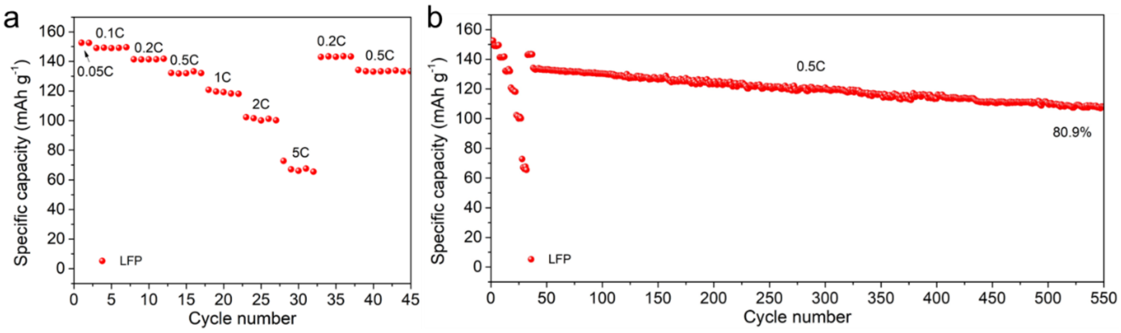

Supplementary Fig. 29. The a) rate performance and b) the cycling performance of the LFP cathode.

Supplementary Table 1. M-Li site distance (0.1 C 2.1 V)

0.1 C 2.1 V	L1 (Å)	L2 (Å)	L3 (Å)	L4 (Å)
A	2.21	3.22	3.32	2.41
B	2.21	3.32	3.32	2.21
C	2.21	3.32	3.32	2.21
D	2.52	2.72	3.12	2.72
E	2.31	2.72	3.22	2.72
F	2.31	3.32	3.22	2.21

Supplementary Table 2. M-Li site distance (0.1 C 1.7 V)

0.1 C 1.7 V	L1 (Å)	L2 (Å)	L3 (Å)	L4 (Å)
A	2.11	3.31	3.51	2.51
B	2.41	2.71	3.21	2.91
C	2.71	2.71	3.01	2.91
D	/	/	/	/
E	2.91	2.81	2.81	2.91
F	2.81	2.81	2.91	2.81

Supplementary Table 3. M-Li site distance (0.1 C 1 V)

0.1 C 1 V	L1 (Å)	L2 (Å)	L3 (Å)	L4 (Å)
A	2.40	2.80	2.90	2.90
B	2.70	2.21	3.00	3.41
C	2.90	2.31	2.90	3.31
D	2.50	3.21	3.00	2.71
E	3.21	2.71	2.50	2.91
F	3.21	2.51	2.50	3.31

Supplementary Table 4. M-Li site distance (20 C 2.1 V)

20 C 2.1 V	L1 (Å)	L2 (Å)	L3 (Å)	L4 (Å)
A	2.56	2.45	3.10	3.27
B	/	/	/	/
C	/	/	/	/
D	2.96	3.48	2.85	2.45
E	3.06	3.68	2.85	2.54
F	/	/	/	/

Supplementary Table 5. M-Li site distance (20 C 1.7 V)

20 C 1.7 V	L1	L2	L3	L4
A	2.21	2.82	3.61	2.92
B	2.52	2.92	3.12	2.82
C	2.41	2.82	3.42	2.82
D	/	/	/	/
E	2.51	2.82	3.01	2.82
F	2.52	2.82	3.22	2.82

Supplementary Table 6. M-Li site distance (20 C 1 V)

20 C 1 V	L1 (Å)	L2 (Å)	L3 (Å)	L4 (Å)
A	2.40	2.71	3.11	3.01
B	2.51	2.71	3.11	2.91
C	2.81	2.81	2.81	3.01
D	2.91	2.71	2.81	3.01
E	2.91	2.81	2.91	3.01
F	2.91	2.71	2.91	2.91

Supplementary Table 7. Comparison of rate performance in different literatures.

Materials	Voltage range (V vs. Li^+/Li)	Specific capacity	Rate capability	Ref.
**$Nb_{16}W_5O_{55}$/rGO**	1.0-3.0	241.1 mAh g^{-1} at 0.2C	116.1 mAh g^{-1} at 80C	Our work
**$Nb_{16}W_5O_{55}$**	1.0-3.0	234.8 mAh g^{-1} at 0.2C	53.8 mAh g^{-1} at 80C	Our work
**$Nb_{16}W_5O_{55}$**	1.0-3.0	225 mAh g^{-1} at 0.2C	58.4 mAh g^{-1} at 60C	2
**$Nb_{18}W_{16}O_{93}$**	1.0-3.0	220 mAh g^{-1} at 0.2C	70 mAh g^{-1} at 100C	2
**$Nb_{18}W_8O_{69}$**	1.0-3.0	265 mAh g^{-1} at 0.5C	155 mAh g^{-1} at 20C	3
**Nb_{16}/Nb_{18}**	1.0-3.0	215.3 mAh g^{-1} at 0.1C	86 mAh g^{-1} at 100C	4
**$Nb_{14}W_3O_{44}$**	1.0-3.0	221.3 mAh g^{-1} at 0.5C	57.7 mAh g^{-1} at 100C	5
**$Nb_{12}WO_{33}$**	1.0-3.2	221 mAh g^{-1} at 1C	142 mAh g^{-1} at 20C	6
**$Nb_{6.7}W_{10.3}O_{47}$**	1.3−3.3	139.43 mAh g^{-1} at 0.5C	111.2 mAh g^{-1} at 20C	7
**Nb_2WO_8**	0.8-2.8	161.56 mAh g^{-1} at 1C	48.7 mAh g^{-1} at 20C	7
**$Nb_2W_3O_{14}$**	1.2-3.0	150 mAh g^{-1} at 0.1C	59.4 mAh g^{-1} at 60C	8
**$Ti_2Nb_{10}O_{29}$**	1.0-2.4	311 mAh g^{-1} at 0.1C	50 mAh g^{-1} at 50C	9
**$TiNb_{24}O_{62}$**	1.0-3.0	240 mAh g^{-1} at 0.1C	100 mAh g^{-1} at 15C	10
**$Ce_{0.01}$-$TiNb_2O_7$**	1.0-3.0	323.1 mAh g^{-1} at 0.2C	227.2 mAh g^{-1} at 40C	11
**Cr^{3+}-doped-$Ti_2Nb_{10}O_{29}$**	1.0–2.5	310 mAh g^{-1} at 1C	220 mAh g^{-1} at 40C	12
**$FeNb_{11}O_{29}$ nanotubes**	1.0-3.0	272.6 mAh g^{-1} at 0.1C	102.8 mAh g^{-1} at 20C	13
**PNb_9O_{25}**	1.0-3.0	230 mAh g^{-1} at 0.1C	30 mAh g^{-1} at 60C	14
**VNb_9O_{25}**	1.0-3.0	177 mAh g^{-1} at 0.1C	0 mAh g^{-1} at 60C	14
**$Mo_{1.5}W_{1.5}Nb_{14}O_{44}$**	1.0-3.0	218.1 mAh g^{-1} at 1C	92 mAh g^{-1} at 100C	15
**$(W_{0.2}V_{0.8})_3O_7$**	1.0-3.2	283 mAh g^{-1} at 0.2C	80 mAh g^{-1} at 20C	16
**$CeNb_3O_9$**	0.8-3.0	195 mAh g^{-1} at 0.5C	103 mAh g^{-1} at 60C	17
**$Mo_3Nb_2O_{14}$**	1.5-3.0	225 mAh g^{-1} at 0.1C	24 mAh g^{-1} at 60C	8
**T-Nb_2O_5**	1.0-2.8	157.8 mAh g^{-1} at 7C	80.0 mAh g^{-1} at 70C	18
**H-Nb_2O_5**	1.2-3.0	221 mAh g^{-1} at 0.1C	25 mAh g^{-1} at 5C	19
**$KNb_6O_{15}F$**	1.0-3.0	150 mAh g^{-1} at 0.5C	80 mAh g^{-1} at 20C	20
		**Surface engineering**		
**$TiNb_2O_7$@C**	1.0-3.0	243.6 mAh g^{-1} at 0.1C	100 mAh g^{-1} at 40C	21
**$Nb_{18}W_{16}O_{93}$/C**	1.0-3.0	225.7 mAh g^{-1} at 0.2C	138 mAh g^{-1} at 20C	22
**$Ti_2Nb_{10}O_{29-x}$@C**	1.0-2.5	310 mAh g^{-1} at 1C	197 mAh g^{-1} at 20C	23
**H-Nb_2O_5/C**	1.2-3.0	230.7 mAh g^{-1} at 0.5C	136.5 mAh g^{-1} at 30C	24

Supplementary Table 8. Analysis of EIS fitting errors.

	$rGO/Nb_{16}W_5O_{55}$		$Nb_{16}W_5O_{55}$	
	**Value**	**Error (%)**	**Value**	**Error (%)**
**R_{ohm} (ohm)**	3.178	1.38	21.38	0.70
**R_{SEI} (ohm)**	10.32	5.97	45.30	5.46
**CPE1-T**	1.2199E-5	11.93	2.6982E-5	5.64
**CPE1-P**	0.99421	2.83	0.81995	1.38
**R_{ct} (ohm)**	2.976	9.78	19.65	10.23
**CPE2-T**	1.1115E-5	13.05	5.6762E-3	10.56
**CPE2-P**	0.98657	4.34	0.50886	10.03
**W-R**	88	7.45	9.229	4.53
**W-T**	47.04	12.60	0.027939	4.82
**W-P**	0.3188	2.78	0.47562	0.359

Supplementary Table 9. Comparison of specific power density and energy density in different literatures.

Materials	Voltage range (V vs. Li^+/Li)	Specific power density (W kg^{-1})	Specific energy density (Wh kg^{-1})	Ref.
**$Nb_{16}W_5O_{55}$/rGO**	1.0-3.0	14860.8	185.76	Our work
**$Nb_{16}W_5O_{55}$**	1.0-3.0	6886.4	86.08	Our work
**$Nb_{16}W_5O_{55}$**	1.0-3.0	5606.4	93.44	2
**$Nb_{18}W_{16}O_{93}$**	1.0-3.0	11200	112	2
**$Nb_{18}W_8O_{69}$**	1.0-3.0	4960	248	3
**Nb_{16}/Nb_{18}**	1.0-3.0	13760	137.6	4
**$Nb_{14}W_3O_{44}$**	1.0-3.0	9232	92.32	5
**Nb_{14} W_3O_{44}**	1.0-3.0	4105.6	205.28	7
**$Nb_{14}W_3O_{44}$ nanowires**	1.0-3.0	1172.368	249.44	25
**nano-sized $Nb_{14}W_3O_{44}$**	1.0-3.0	16624	166.24	26
**$Nb_{12}WO_{33}$**	1.0-3.2	4544	227.2	6
**$Nb_{6.7}W_{10.3}O_{47}$**	1.3–3.3	3558.4	177.92	7
**Nb_2WO_8**	0.8-2.8	1558.4	77.92	7
**$Nb_2W_3O_{14}$**	1.2-3.0	5702.4	95.04	8

Supplementary References

1 Kocer, C. P., Griffith, K. J., Grey, C. P. & Morris, A. J. Cation Disorder and Lithium Insertion Mechanism of Wadsley-Roth Crystallographic Shear Phases from First Principles. *J. Am. Chem. Soc.* **141**, 15121-15134, (2019).

2 Griffith, K. J., Wiaderek, K. M., Cibin, G., Marbella, L. E. & Grey, C. P. Niobium tungsten oxides for high-rate lithium-ion energy storage. *Nature* **559**, 556-563, (2018).

3 Griffith, K. J. & Grey, C. P. Superionic Lithium Intercalation through 2 × 2 nm^2 Columns in the Crystallographic Shear Phase $Nb_{18}W_8O_{69}$. *Chem. Mater.* **32**, 3860-3868, (2020).

4 Ma, J. *et al.* Regulating the local coordination model of homologous and heterogeneous niobium tungsten oxides toward ultrafast lithium storage. *Energy Stor. Mater.* **63**, 102979, (2023).

5 Yang, Y. *et al.* Achieving Ultrahigh-Rate and High-Safety Li^+ Storage Based on Interconnected Tunnel Structure in Micro-Size Niobium Tungsten Oxides. *Adv. Mater.* **32**, e1905295, (2020).

6 Saritha, D., Pralong, V., Varadaraju, U. V. & Raveau, B. Electrochemical Li insertion studies on $WNb_{12}O_{33}$—A shear ReO_3 type structure. *J. Solid State Chem.* **183**, 988-993, (2010).

7 Yao, W. *et al.* Structural Insights into the Lithium Ion Storage Behaviors of Niobium Tungsten Double Oxides. *Chem. Mater.* **34**, 388-398, (2021).

8 Luo, Y. *et al.* Structure and Electrochemical Properties of Bronze Phase Materials Containing Two Transition Metals. *Chem. Mater.* **35**, 8675-8685, (2023).

9 Cheng, Q. *et al.* Bulk $Ti_2Nb_{10}O_{29}$ as long-life and high-power Li-ion battery anodes. *J. Mater. Chem. A* **2**, 17258-17262, (2014).

10 Griffith, K. J., Senyshyn, A. & Grey, C. P. Structural Stability from Crystallographic Shear in TiO_2–Nb_2O_5 Phases: Cation Ordering and Lithiation Behavior of $TiNb_{24}O_{62}$. *Inorg Chem* **56**, 4002-4010, (2017).

11 Shi, A. *et al.* Highly oxidized state dopant induced Nb-O bond distortion of $TiNb_2O_7$ for extremely fast-charging batteries. *Nano Energy* **123**, 109349, (2024).

12 Deng, S. *et al.* Synergy of Ion Doping and Spiral Array Architecture on $Ti_2Nb_{10}O_{29}$: A New Way to Achieve High-Power Electrodes. *Adv. Funct. Mater.* **30**, 2002665, (2020).

13 Zheng, R. *et al.* $FeNb_{11}O_{29}$ nanotubes: Superior electrochemical energy storage performance and operating mechanism. *Nano Energy* **58**, 399-409, (2019).

14 Preefer, M. B. *et al.* Multielectron Redox and Insulator-to-Metal Transition upon Lithium Insertion in the Fast-Charging, Wadsley-Roth Phase PNb_9O_{25}. *Chem. Mater.* **32**, 4553-4563, (2020).

15 Tao, R. *et al.* Insight into the Fast-Rechargeability of a Novel $Mo_{1.5}W_{1.5}Nb_{14}O_{44}$ Anode Material for High-Performance Lithium-Ion Batteries. *Adv. Energy Mater.* **12**, 2200519, (2022).

16 Wyckoff, K. E. *et al.* High-Capacity Li^+ Storage through Multielectron Redox in the Fast-Charging Wadsley–Roth Phase $(W_{0.2}V_{0.8})_3O_7$. *Chem. Mater.* **32**, 9415-9424, (2020).

17 Yang, L. *et al.* Atomic Short-Range Order in a Cation-Deficient Perovskite Anode for Fast-Charging and Long-Life Lithium-Ion Batteries. *Adv. Mater.* **34**, 2200914, (2022).

18 Han, H. *et al.* Li iontronics in single-crystalline T-Nb_2O_5 thin films with vertical ionic transport channels. *Nat. Mater.* **22**, 1128–1135, (2023).

19 Griffith, K. J., Forse, A. C., Griffin, J. M. & Grey, C. P. High-Rate Intercalation without Nanostructuring in Metastable Nb_2O_5 Bronze Phases. *J. Am. Chem. Soc.* **138**, 8888-8899, (2016).

20 Cao, D., Yao, Z., Liu, J., Zhang, J. & Li, C. H-Nb_2O_5 wired by tetragonal tungsten bronze related domains as high-rate anode for Li-ion batteries. *Energy Stor. Mater.* **11**, 152-160, (2018).

21 Wu, D. *et al.* Realizing rapid electrochemical kinetics of Mg^{2+} in Ti-Nb oxides through a Li^+ intercalation activated strategy toward extremely fast charge/discharge dual-ion batteries. *Energy Stor. Mater.* **52**, 94-103, (2022).

22 Liu, W., Xu, M. & Zhu, M. Design of a niobium tungsten oxide/C micro-structured electrode for fast charging lithium-ion batteries. *Inorganic Chemistry Frontiers* **8**, 3998-4005, (2021).

23 Deng, S. *et al.* Boosting fast energy storage by synergistic engineering of carbon and deficiency. *Nat. Commun.* **11**, 132, (2020).

24 Meng, J. *et al.* Identification of Phase Control of Carbon-Confined Nb_2O_5 Nanoparticles toward High-Performance Lithium Storage. *Adv. Energy Mater.* **9**, 1802695, (2019).

25 Yan, L. *et al.* $W_3Nb_{14}O_{44}$ nanowires: Ultrastable lithium storage anode materials for advanced rechargeable batteries. *Energy Stor. Mater.* **16**, 535-544, (2019).

26 Guo, C. *et al.* Nano-Sized Niobium Tungsten Oxide Anode for Advanced Fast-Charge Lithium-Ion Batteries. *Small* **18**, e2107365, (2022).
